# Diffusion-weighted MRI and ^18^F-FDG PET/CT in assessing the response to neoadjuvant chemoradiotherapy in locally advanced esophageal squamous cell carcinoma

**DOI:** 10.1186/s13014-021-01852-z

**Published:** 2021-07-19

**Authors:** Xin Xu, Zhi-Yong Sun, Hua-Wei Wu, Chen-Peng Zhang, Bin Hu, Ling Rong, Hai-Yan Chen, Hua-Ying Xie, Yu-Ming Wang, Hai-Ping Lin, Yong-Rui Bai, Qing Ye, Xiu-Mei Ma

**Affiliations:** 1 Department of Radiation Oncology, Ren Ji Hospital, School of Medicine, Shanghai Jiaotong University, 160 Pujian Road, Shanghai, 200127 China; 2 Department of Thoracic Surgery, Ren Ji Hospital, School of Medicine, Shanghai Jiaotong University, 160 Pujian Road, Shanghai, 200127 China; 3 Department of Radiology, Ren Ji Hospital, School of Medicine, Shanghai Jiaotong University, Shanghai, China; 4 Department of Nuclear Medicine, Ren Ji Hospital, School of Medicine, Shanghai Jiaotong University, Shanghai, China

**Keywords:** Esophageal squamous cell cancer, Neoadjuvant chemoradiotherapy, Pathological complete response, ^18^F-FDG PET/CT, DW-MRI

## Abstract

**Background:**

Neoadjuvant chemoradiotherapy (nCRT) followed by surgery is a currently widely used strategy for locally advanced esophageal cancer (EC). However, the conventional imaging methods have certain deficiencies in the evaluation and prediction of the efficacy of nCRT. This study aimed to explore the value of functional imaging in predicting the response to neoadjuvant chemoradiotherapy (nCRT) in locally advanced esophageal squamous cell carcinoma (ESCC).

**Methods:**

Fifty-four patients diagnosed with locally advanced ESCC from August 2017 to September 2019 and treated with nCRT were retrospectively analyzed. DW-MRI scanning was performed before nCRT, at 10–15 fractions of radiotherapy, and 4–6 weeks after the completion of nCRT. ^18^F-FDG PET/CT scans were performed before nCRT and 4–6 weeks after the completion of nCRT. These ^18^F-FDG PET/CT and DW-MRI parameters and relative changes were compared between patients with pathological complete response (pCR) and non-pCR.

**Results:**

A total of 8 of 54 patients (14.8%) were evaluated as disease progression in the preoperative assessment. The remaining forty-six patients underwent operations, and the pathological assessments of the surgical resection specimens demonstrated pathological complete response (pCR) in 10 patients (21.7%) and complete response of primary tumor (pCR-T) in 16 patients (34.8%). The change of metabolic tumor volume (∆MTV) and change of total lesion glycolysis (∆TLG) were significantly different between patients with pCR and non-pCR. The SUVmax-T_post_, MTV-T_post_, and TLG-T_post_ of esophageal tumors in ^18^F-FDG PET/CT scans after neoadjuvant chemoradiotherapy and the ∆ SUVmax-T and ∆MTV-T were significantly different between pCR-T versus non-pCR-T patients. The esophageal tumor apparent diffusion coefficient (ADC) increased after nCRT; the ADC_during_, ADC_post_ and ∆ADC_during_ were significantly different between pCR-T and non-pCR-T groups. ROC analyses showed that the model that combined ADC_during_ with TLG-T_post_ had the highest AUC (0.914) for pCR-T prediction, with 90.0% and 86.4% sensitivity and specificity, respectively.

**Conclusion:**

^18^F-FDG PET/CT is useful for re-staging after nCRT and for surgical decision. Integrating parameters of ^18^F-FDG PET/CT and DW-MRI can identify pathological response of primary tumor to nCRT more accurately in ESCC.

## Background

Esophageal cancer (EC) is the seventh most frequently diagnosed cancers and the sixth leading causes of cancer death worldwide [1]. It is one of the most common malignancy in China, with the third highest morbidity and mortality rate [2]. More than 90% of patients with EC in China have esophageal squamous cell carcinoma (ESCC). The CROSS study established the position of neoadjuvant chemoradiotherapy for esophageal cancer treatment [3]. Neoadjuvant chemoradiotherapy (nCRT) followed by surgery is currently widely used strategy for locally advanced EC. The NEOCRTEC5010 study showed that patients with ESCC were more likely to benefit from nCRT with higher pathological complete response (pCR) and better survival [4].

Many studies have revealed that patients who achieved pCR after nCRT had the most favorable survival [5, 6]. For the pathological complete responders, active surveillance or definitive chemoradiotherapy might be an alternative choice which can preserve organ function instead of esophagectomy, but the outcome of this strategy still needs further research to confirm [7]. For patients with obvious residual tumor or progression after nCRT, the benefits from nCRT are limited [8]. Thus, early surgical intervention might be required or alternative neoadjuvant treatment methods for these patients should be explored, neoadjuvant immunotherapy, for example. Therefore, it is very important to accurately assess and predict the efficacy of neoadjuvant chemoradiotherapy and that can help us understand the prognosis of patients in time and perform better treatment strategies.

At present, conventional imaging methods have certain defects (focus only on the volume change) in the evaluation of the efficacy of nCRT. Whereas functional imaging can more comprehensively reflect the biological and microstructural characterization of tumors. The changes of these aspects of tumors can be observed earlier than volumetric changes of tumors. The use of the ^18^F-FDG tracer in PET/CT allows for an assessment of cellular glucose metabolism in tumors by measuring the standardized uptake value (SUV) that can be complementary in discriminating treatment response [9, 10]. Diffusion-weighted imaging (DWI) is a functional magnetic resonance imaging technique that enables detection of Brownian motion of water protons in tissues and a quantitative measure of tissue micro-environment. More recently, several studies showed the apparent diffusion coefficient (ADC) measured using DWI might be useful in predicting pathological response to nCRT in esophageal cancer before imaging volume change occurred [11, 12].

Integrating ^18^F-PET/CT and DW-MRI may provide comprehensive information for cancer response [13, 14]. Therefore, the aim of the present study was to evaluate the value of ^18^F-FDG PET/CT and DW-MRI to assess and predict pathological response in patients who underwent nCRT for ESCC.

## Methods

### Patients

Patients with biopsy and imaging proved local advanced esophageal squamous cell cancer who were considered eligible for neoadjuvant chemoradiotherapy followed by esophagectomy were retrospectively analyzed. All the patients were cT3-4N0M0 or cT1-4N1-2M0 [15]. The study was approved by the Medical Ethics Committee of our institute and all the patients provided written informed consent.

### Treatment

All the patients received paclitaxel/cisplatin chemotherapy and concurrent radiotherapy. Each patient received radiation of 41.4 Gy/23 fractions complied by intensity modulated radiotherapy or volumetric modulated arc therapy. The gross tumor volume (GTV) was defined as the primary tumor and suspected metastatic lymph nodes determined by enhanced CT and PET/CT. Elective nodal irradiation was used in our study. The clinical target volume (CTV) included 3 cm expansion of GTV to proximal and distal margins and 5 mm towards radial direction, and also included the regional lymph nodes which were prophylactic irradiated. Planning target volume (PTV) was created from CTV by adding a uniform margin of 5 mm. One of two regimens was selected for concomitant chemotherapy, including 1. 3-weekly regimen: paclitaxel 135 mg/m^2^ + cisplatin 75 mg/m^2^ every 3 weeks for 2 cycles; 2. weekly regimen: paclitaxel 45 mg/m^2^ + cisplatin 25 mg/m^2^ weekly for 5 cycles. Approximately 4 to 6 weeks after the completion of chemoradiotherapy, patients underwent clinical re-staging. Patients without disease progression (non-PD) were scheduled for surgery and patients who had progressive disease did not proceed to surgery but the second-line systemic treatment. Surgery was performed 6 to 8 weeks after completion of chemoradiotherapy. A minimal invasive McKeown or Ivor Lewis esophagectomy was performed, including two-field lymphadenectomy or three-filed lymphadenectomy in individual patients.

### Pathologic assessment

Pathological examination of the surgical resection specimen was assessed by pathologists who were blinded to the results of ^18^F-FDG PET/CT and DW-MRI scans. Patients were staged in accordance with the 7th edition of the Union for International Cancer Control (UICC) [15]. Pathological complete response (pCR) was defined as the absence of residual tumor cells in the primary site and resected lymph nodes. Complete response of primary tumor (pCR-T) was defined as no evidence of residual tumor cells in the primary site.

### Image acquisition and analysis

Patients underwent ^18^F-FDG PET/CT scanning at 2 time points, before nCRT (PET/CT_pre_) and 4–6 weeks after the completion of nCRT before surgery (PET/CT_post_). PET was performed with a dedicated whole-body PET/CT scanner (Biograph mCT, Siemens Systems). All patients fasted for at least 6 h before PET scan and demonstrated blood glucose levels lower than 7 mmol/L at the time of injection. The dose of ^18^F-FDG injection was 0.1 mCi/kg of body weight. The PET/CT scan began 45–60 min after the 18F-FDG injection. An unenhanced CT image was obtained from the base of the skull to midthigh using 120 kV, 140 mA, and slice thickness of 5.0 mm. Immediately after CT, PET was performed covering the same field. The acquisition time for PET was 4–5 min per table position. Patients were instructed to breathe shallowly during the acquisition of CT and PET. Two radiologists blinded to the clinical data independently analyzed the PET/CT imaging. A radiologist first identified the approximate center of the tumor then the software automatically drew a region of interest (ROI). The primary lesion of the esophagus with adjacent para-esophageal lymph nodes (T) and the regional lymph nodes (LN) were contoured separately. The software automatically calculated SUVmax, SUVmean, and metabolic tumor volume (MTV). Total lesion glycolysis (TLG) = SUVmean × MTV, ΔSUV = (SUV_post_ − SUV_pre_)/SUV_pre,_ ΔMTV = (MTV_post_ − MTV_pre_)/MTV_pre_, ΔTLG = (TLG_post_ − TLG_pre_)/TLG_pre_.

Patients underwent MRI scanning at three time points, including before nCRT (MRI_pre_), 2–3 weeks (10–15 fraction) during nCRT (MRI_during_), and 4–6 weeks after the completion nCRT (MRI_post_). A Philips Intera 3.0 T MRI was used. All patients were scanned in the supine position and were trained before the examination to take shallow slow breaths. The transverse scanning range only included esophageal primary lesions. Sagittal and transverse T2-weighted images were obtained. The parameters for the upper and middle lesions included a 1100–1300 ms repetition time (TR), 90 ms echo time (TE), 4 mm thickness, 0.4 mm spacing, 323 × 215 matrix, and 2.00 incentive times (NEX). The parameters for the lower lesions included 1336 ms TR, 90 ms TE, 3 mm thickness, 0.5 mm spacing, 280 × 245 matrix, and 2.00 NEX. DW-MRIs were then obtained by a single-shot spin echo echo-planar imaging (SE-EPI) sequence with *b* values of 0 and 800 s/mm2 (1067 ms TR, 66 ms TE, 3 mm thickness, 0.3 mm spacing, 132 × 114 matrix size, and 2.0 NEX). All original DW-MRIs were transferred to a workstation (Philips Intellispace Portal v6.0) to create ADC maps. Two experienced radiologists blinded to the clinical data were responsible for the interpretation of the images. The lesion location was determined on the T2-weighted images. An axial slice of the DWI that showed the most predominant tumor size corresponding to T2-weighted images was selected. The region of interest (ROI) was contoured manually and encompassed the entire essence of the tumor displayed on the ADC map, excluding areas of necrotic, cystic, or hemorrhagic change. ΔADC_during_ = (ADC_during_ − ADC_pre_)/ADC_pre_, ΔADC_post_ = (ADC_post_ − ADC_pre_)/ADC_pre_.

### Statistical analysis

The Mann–Whitney U test was used to compare the parameters of ^18^F-FDP PET/CT and DW-MRI between patients with different responses. Logistic regression was used to create a combined predicting model. All tests were performed using SPSS 26.0. The diagnostic performance for the detection of complete response was calculated by receiver operator characteristic (ROC) curve analysis. The areas under the curve (AUCs) and the diagnostic performance measures sensitivity and specificity were calculated by using MedCal software. A statistically significant difference was identified when *p* value was < 0.05 (two-sided).

## Results

### Patients’ characteristics and treatment outcomes

**Fig. 1:**
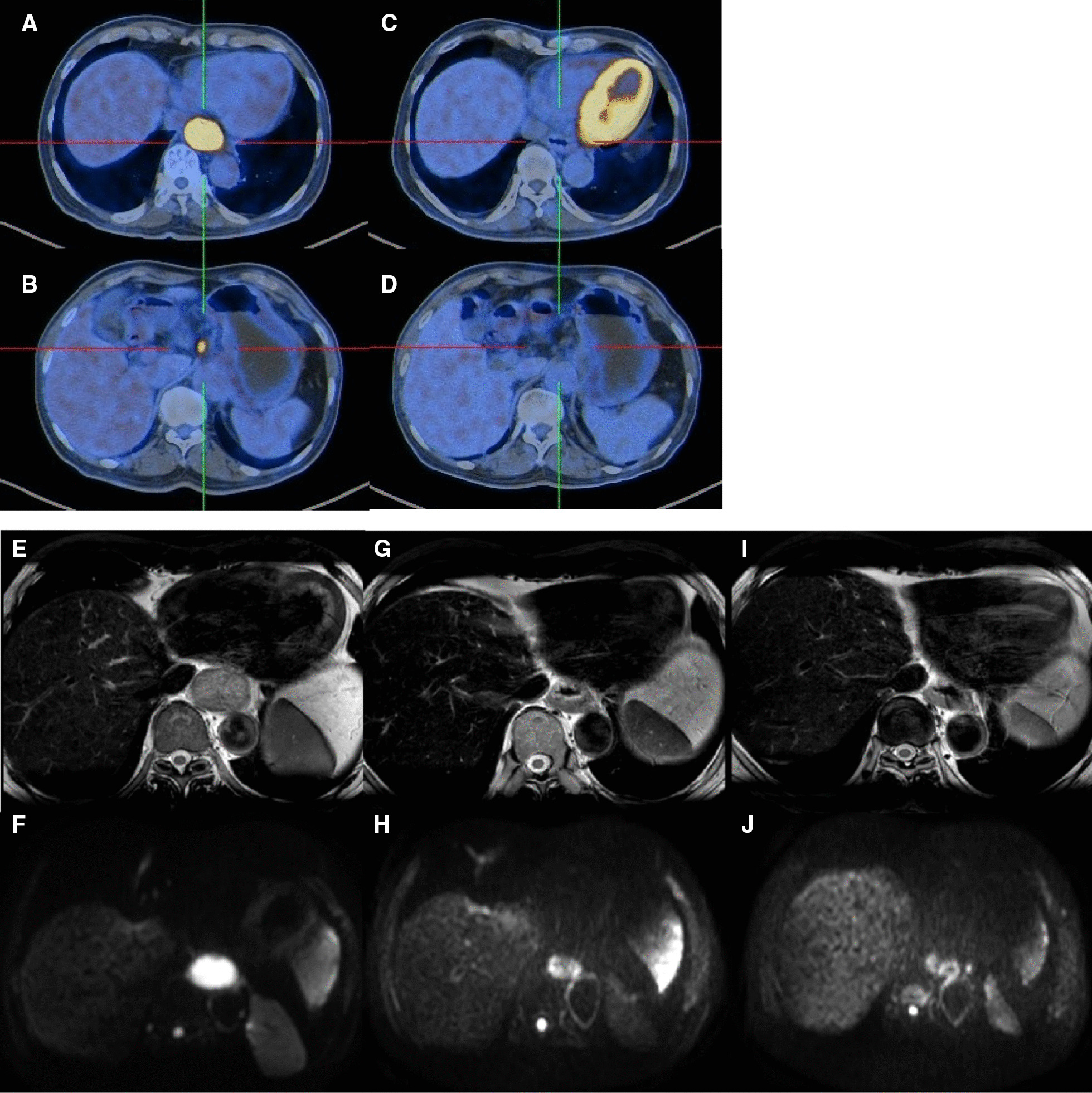
Images from a patient with a cT3N1M0 lower thoracic esophageal squamous cell carcinoma with pathological complete response to neoadjuvant chemoradiotherapy. **A**, **B**: PET/CT images before nCRT; **C**, **D**: PET/CT images five weeks after nCRT; **E**, **G**, **I**: T2 weighted MRI images, **F**, **H**, **J**: DW-MRI images (b value = 800 s/mm^2^); **E**, **F**: before nCRT; **G**, **H**: 13 fractions during nCRT; **I**, **J**: five weeks after nCRT. *PET/CT* positron emission tomography/computed tomography; *DW-MRI* diffusion-weighted magnetic resonance imaging

A total of 66 consecutive patients newly diagnosed locally advanced esophageal cancer underwent neoadjuvant chemoradiotherapy at our Institute between August 2017 and September 2019. A total of 12 patients were excluded from the analysis because, six patients had neither ^18^F-FDG PET/CT nor DW-MRI examination, one patient withdrew after neoadjuvant chemoradiotherapy and five patients refused surgery. The remaining 54 patients were eligible for analysis. A total of 51 patients had PET/CT scans before and after nCRT and 43 patients had MRI scans before, during and after nCRT. The baseline characteristics of these patients are outlined in Table 1. A total of 8/54 patients (14.8%) demonstrated disease progression during preoperative assessment. Forty-six patients underwent operation, and the pathological assessments of surgical resection specimens demonstrated pathological complete response (pCR) in 10 patients (21.7%) and complete response of primary tumor (pCR-T) in 16 patients (34.8%). 29 patients were pathological negative of lymph node and 17 patients were positive including 13 patients with pN1 and 4 patients with pN2. No significant differences were observed in age, tumor location, tumor length, clinical stages, or chemotherapy regimens between patients with pCR and non-pCR. Figure 1 showed ^18^F-FDG-PET/CT and DW-MRI images from a patient with pCR to nCRT.

**Table 1:** Clinical characteristics of the study population

Characteristics	All (n = 54)	pCR (n = 10)	Non-pCR (n = 36)	*P*
	n (%)	n (%)	n (%)	
Age				0.67
Mean(range)	63 (48,75)	63 (55,70)	62 (48,75)	
≤ 60	15 (27.8)	2 (20.0)	12 (33.3)	
> 60	39 (72.2)	8 (80.0)	24 (66.7)	
Sex				0.50
Male	49 (90.7)	10 (100.0)	31 (86.1)	
Female	5 (9.3)	0 (0.0)	5 (13.9)	
Tumor location				0.28
Proximal	8 (14.8)	2 (20.0)	5 (13.8)	
Middle	16 (29.6)	1 (10.0)	13 (36.2)	
Distal	30 (55.5)	7 (70.0)	18 (50.0)	
Length, cm				0.47
Mean (range)	6 (3,10)	6 (4,9)	6 (3,10)	
≤ 6 cm	38 (70.4)	6 (60.0)	28 (77.8)	
> 6 cm	16 (29.6)	4 (40.0)	8 (22.2)	
Clinical T stage				0.39
T1-2	2 (3.7)	1 (10.0)	1 (2.8)	
T3-4	52 (96.3)	9 (90.0)	35 (97.2)	
Clinical N stage				0.61
N negative	3 (48.1)	1 (60.0)	2 (50.0)	
N positive	51 (51.9)	9 (40.0)	34 (50.0)	
Clinical stage				0.91
II	2 (3.7)	1 (10.0)	1 (2.8)	
III	52 (96.3)	9 (90.0)	35 (97.2)	
Chemotherapy				
Weekly	32 (59.3)	4 (40.0)	21 (58.3)	0.50
3-weekly	22 (40.7)	6 (60.0)	15 (41.7)	

### ^18^F-FDG PET/CT for re-staging and predicting pCR of all lesions

In the ^18^F-FDG-PET/CT scans before operation, eight patients showed disease progression. In these patients, three patients displayed multiple distant metastases, one patient had a single anterior serratus muscle metastasis confirmed by biopsy, one patient showed an enlarged lymph node with corresponding increased uptake of FDG, and three patients demonstrated newly diagnosed lymph nodes (one case with supraclavicular lymph node metastasis and two cases with para-aortic lymph node metastasis).

On the restaging PET/CT, 26 patients had positive regional lymph nodes (SUV higher than the normal tissues of mediastinum, usually higher than 2.5) and 13 of them had pathologically confirmed positive lymph nodes. The sensitivity and specificity of PET/CT for pN + staging was 76.5% and 51.9% respectively. A total of 30 pathological positive lymph nodes from 17 patients were detected in surgical specimens, in which 11 pathological positive lymph nodes from seven patients had not been identified by either baseline PET/CT or preoperative PET/CT.

The pre- and post-nCRT ^18^F-FDG PET/CT parameters SUVmax, MTV and TLG of total lesions were analysed. These parameters of both pre- and post-nCRT ^18^F-FDG PET/CT were not significantly different between pCR and non-pCR group (*p* > 0.05). However, the changes in MTV and TLG from baseline ^18^F-FDG PET/CT scans to scans acquired after nCRT were significantly different between patients with pCR and non-pCR [∆MTV (median, IQR): − 0.90 (− 0.86, − 0.94) for pCR versus − 0.78 (− 0.59, − 0.83) for non-pCR, *p* = 0.00, and ∆TLG (median, IQR): − 0.97 (− 0.92, − 0.99) for pCR versus − 0.90 (− 0.86, − 0.94) for non-pCR, *p* = 0.01]. ROC analyses for ∆MTV and ∆TLG resulted in AUCs of 0.816 and 0.787, respectively, for discriminating pCR from non-pCR. Using 0.84 as cut-off value of ∆MTV, 16 patients were evaluated as clinical complete response (cCR) and 8 in which were pCR. The sensitivity and specificity of ∆MTV for predicting pCR of total lesion was 88.9% and 77.1% respectively.

### ^18^F-FDG PET/CT and DW-MRI parameters for predicting pCR of esophageal primary tumor

The relationship between the ^18^F-FDG PET/CT parameters in esophagus tumors and pathological complete response of primary tumors (pCR-T) are shown in Table 2. The SUVmax, MTV, and TLG in the ^18^F-FDG PET/CT scans after neoadjuvant chemoradiotherapy were significantly different between pCR-T versus non-pCR-T patients [SUVmax_post_ (median, IQR): 4.19 (3.28, 4.73) for pCR-T versus 5.65 (3.99,8.76) for non-pCR-T, *p* = 0.02, MTV_post_ (median, IQR): 2.28 (1.15, 3.55) for pCR–T versus 5.23 (2.94, 6.89) for non-pCR-T, *p* = 0.00, and TLG_post_ (median, IQR): 6.97 (3.74,11.62) for pCR–T versus 17.13 (9.09,28.30) for non-pCR-T, *p* = 0.00]. The changes in SUVmax and MTV from baseline ^18^F-FDG PET/CT scans were significantly different between patients with pCR-T versus non- pCR-T, but ∆TLG values were not significantly different between patients with pCR-T versus non- pCR-T.

**Table 2:** ^18^F-FDG PET/CT parameters between patients with pCR-T and non-pCR-T

Parameters	pCR-T (n = 15)	non-pCR-T (n = 29)	P value
	Mean (IQR)		
SUVmax-T_pre_	20.76(16.82,25.63)	19.94(16.82,25.13)	0.78
MTV-T_pre_	18.06(10.95,36.03)	22.44(14.21,29.58)	0.59
TLG-T_pre_	173.42(64.96,339.75)	196.60(116.30,270.60)	0.59
SUVmax-T_post_	4.19(3.28,4.73)	5.65(3.99,8.76)	***0.02***
MTV-T_post_	2.28(1.15,3.55)	5.23(2.94,6.89)	***0.00***
TLG-T_post_	6.97(3.74,11.62)	17.13(9.09,28.30)	***0.00***
ΔSUVmax-T	− 0.80(− 0.76, − 0.85)	− 0.70(− 0.58, − 0.79)	***0.01***
ΔMTV-T	− 0.90(− 0.66, − 0.95)	− 0.77(− 0.63, − 0.86)	***0.04***
ΔTLG-T	− 0.96(− 0.81, − 0.99)	− 0.89(− 0.87, − 0.95)	0.07

P values less than 0.05 are indicated in bold italics

^*18*^*F-FDG-PET/CT* ^18^F-Fluorodeoxyglucose-positron emission tomography/computed tomography; *pCR-T* complete response of primary tumor; *SUVmax* maximum standard uptake value; *MTV* metabolic tumor volume; *TLG* total lesion glycolysis

The primary tumor ADCs of baseline DW-MRI scans were not significantly associated with pathological complete response of primary tumors (pCR-T). Following chemoradiotherapy, the primary tumor ADC increased, the ADC_during_, ADC_post_ and ∆ADC_during_ were significantly different between pCR-T and non-pCR-T groups [ADC_during_ (median, IQR): 2.70 (2.57, 2.93) for pCR-T versus 2.13 (1.71, 2.32) for non- pCR-T, *p* = 0.00, ADC_post_ (median, IQR): 2.82 (2.75, 2.90) for pCR-T versus 2.46 (2.26, 2.84) for non- pCR-T, *p* = 0.02, and ∆ADC_during_ (median, IQR): 0.89 (0.63,1.27) for pCR-T versus 0.48 (0.37, 0.80) for non- pCR-T, *p* = 0.00], however the ∆ADC_post_ was not significantly different between these two groups (Table 3).

**Table 3:** DW-MRI parameters between patients with pCR-T and non-pCR-T

Parameters	pCR-T (n = 12)	non-pCR-T (n = 23)	P value
	Mean (IQR)		
ADC_pre_ (× 10^3^ mm^2^/s)	1.38(1.19, 1.60)	1.38(1.07, 1.57)	0.57
ADC_during_ (× 10^−3^ mm^2^/s)	2.70(2.57, 2.93)	2.13(1.71, 2.32)	***0.00***
ADC_post_ (× 10^−3^ mm^2^/s)	2.82(2.75, 2.90)	2.46(2.26, 2.84)	***0.02***
ΔADC_during_	0.89(0.63,1.27)	0.48(0.37, 0.80)	***0.00***
ΔADC_post_	1.14(0.81,1.29)	0.92(0.61, 1.24)	0.41

P values less than 0.05 are indicated in bold italics

*DW-MRI* diffusion-weighted magnetic resonance imaging; *pCR-T* complete response of primary tumor; *ADC* apparent diffusion coefficient; *IQR* interquartile range

**Fig. 2:**
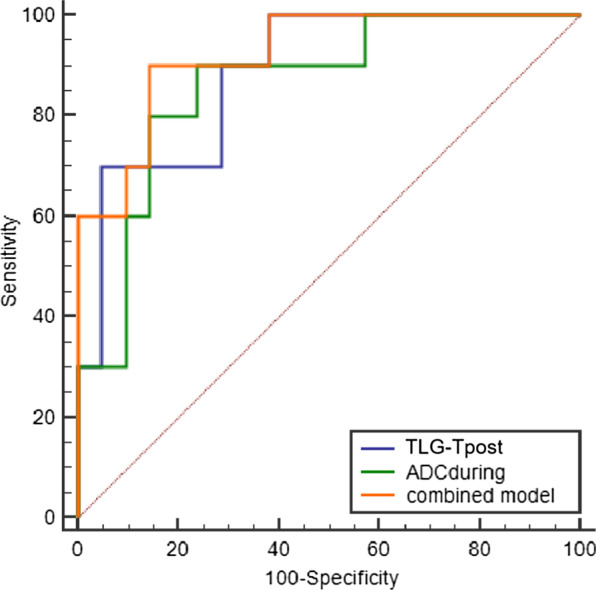
ROC curves of the combined model to predict pCR-T. AUC of TLG-T_post_: 0.828(95% CI: 0.682–0.925); AUC of ADC_during_: 0.880(95%CI: 0.704–0.962); AUC of combined model 0.914(95%CI: 0.759–0.983). *TLG* total lesion glycolysis; *ADC* apparent diffusion coefficient; *ROC* receiver operating characteristic; *AUC* areas under the curve; *CI* confidence interval

For predicting pCR of the esophageal primary tumor, the ROC-AUC value for SUVmax-T_post_, MTV-T_post_, TLG-T_post,_ ΔSUVmax-T, ADC_during_, ADC_post_ and ∆ADC_during_ showed high z statistics with P value < 0.05 (Table 4). To evaluate the complementary value of ^18^F-FDG PET/CT and DW-MRI parameters, we created a logistic regression model for pCR-T prediction. ROC analyses for the ADC_during_ combined with TLG-T_post_ model showed the highest AUC of 0.914 compared with their individual values (Fig. 2). The sensitivity and specificity of the model to predict pathological response of primary tumor was 90.00% and 86.36%, respectively.

**Table 4:** ROC analyses of ^18^F-FDG PET/CT and DW-MRI parameters to predict pCR-T

Parameters	AUC	SE	95% CI	Z statistic	P value	Youden index	Associated criterion	Sensitivity (%)	Specificity (%)
SUVmax-T_post_	0.720	0.0804	0.563–0.846	2.743	***0.0061***	0.4409	≤ 4.42	78.57	65.52
MTV-T_post_	0.819	0.0647	0.672–0.920	4.927	** < *0.0001***	0.5172	≤ 5.076	100.00	51.72
TLG-T_post_	0.828	0.0630	0.682–0.925	5.204	** < *0.0001***	0.5517	≤ 14.65	100.00	55.17
ΔSUVmax-T	0.752	0.0752	0.598–0.869	3.346	***0.0008***	0.4874	≥ 0.7229	86.67	62.07
ΔMTV-T	0.685	0.101	0.528–0.817	1.836	0.0663	0.4299	≥ 0.8937	53.33	89.66
ADC_during_	0.880	0.0602	0.704–0.962	6.325	** < *0.0001***	0.7029	> 2.52	83.33	86.96
ADC_post_	0.771	0.0842	0.588–0.900	3.114	***0.0018***	0.6234	> 2.68	90.91	71.43
ΔADC_during_	0.851	0.0639	0.691–0.949	5.504	** < *0.0001***	0.6522	> 0.53	100.0	65.22
Combined model*	0.914	0.0518	0.759–0.983	7.987	** < *0.0001***	0.7636	≤ 0.475	90.00	86.36

P values less than 0.05 are indicated in bold italics

*Predicted probability of the combined model: P = *e*^(7.325 + 0.244*TLG-T_post_-3.774*ADC_during_)/(1 + *e*^(7.325 + 0.244*TLG-T_post_-3.774*ADC_dur ing_))

*DW-MRI* diffusion-weighted magnetic resonance imaging; ^*18*^*F-FDG-PET/CT* ^18^F-Fluorodeoxyglucose-positron emission tomography/computed tomography; *pCR-T* complete response of primary tumor; *SUVmax* maximum standard uptake value; *MTV* metabolic tumor volume; *TLG* total lesion glycolysis; *ADC* apparent diffusion coefficient; *ROC* receiver operating characteristic; *AUC* areas under the curve; *SE* standard error; *CI* confidence interval

## Discussion

Our study has investigated both ^18^F-PET-CT and DW-MRI to access the histopathological response of nCRT in patients with esophageal squamous cell cancers. The preoperative ^18^F‐FDG PET/CT image is useful in detecting interval metastases, and our quantitative analysis showed that changes of MTV and TLG from baseline FDG-PET/CT images were significantly different between patients with pCR and non-pCR. The preoperative ^18^F‐FDG PET/CT SUVmax, MTV, and TLG of esophageal primary tumors and the changes of SUVmax from baseline FDG PET/CT images were useful for detecting the complete response of esophagus tumors. The ADC value of preoperative DW-MRI could differentiate complete response from non-complete response of esophagus tumors, and the DW-MRI in the first 2–3 weeks of nCRT could early predict this pathological response. The combination model of ADC_during_ and TLG-T_post_ showed the highest AUC for predicting pCR of primary lesion. The present study provides encouraging results for the potential value of multimodal imaging for assessing response to neoadjuvant chemoradiotherapy in esophageal squamous cell cancer.

The role of FDG PET/CT imaging in the detection of interval metastases has been confirmed by most studies [10, 16–19], thus PET/CT were recommended as a restaging tool after nCRT. Our study confirmed that FDG PET/CT imaging was useful for the detection of interval metastases. In our study, 3 of 8 patients with PD presented with multiple interval metastases, and five patients showed single or limited metastases, including some infrequent metastases that were difficult to detect by conventional imaging examination. For these patients, preoperative FDG PET/CT evaluation help to avoid an unnecessary surgery. In previous studies, interval metastases were detected in approximately 8–10% patients after neoadjuvant chemoradiotherapy, however this rate was higher in our study (14%). These results might be due to the later stages of our enrolled patients. In a previous study [18], patients with clinical positive lymph nodes, tumor length > 4 cm, squamous cell carcinoma, and primary tumor baseline SUVmax of > 9.6 showed a higher risk of interval metastases. In addition, these previous studies showed that PET/CT had a high false-positive rate when diagnosing interval metastases. The metastatic lesions detected by PET/CT should be carefully confirmed by histology or other imaging or clinical follow-up. In our study, we also used post-nCRT PET/CT to predict pN + , but the value was limitied, especially the specificity was lower. Pathologic lymph nodes metastases were found frequently even when no evidence on PET/CT findings. It was consistent with the results of some previous studies and might indicate that we need more effective modality for predicting pathological lymph nodes status.

Many clinical trials have been conducted to identify the value of PET parameters to predict pathological response. Several meta-analyses have indicated that ^18^F-FDG PET/CT images were unable to accurately detect pathological response, the sensitivities and specificities were 60%–70% [20–22]. Most of these studies used SUVmax or percentage reduction of SUVmax as criterion. Anonther study indicated that quantitative parameters based on SULmax (maximum standardized uptake values normalized for lean body mass) did not detect TRG3‐4 tumor in 27‐61% of patients [10]. Whereas some previous reports [23, 24] showed that metabolic tumor volume measurements were better predictors of response and survival compared with SUVmax. The same outcome was also found in our research. For all lesions, ∆MTV and ∆TLG were significantly different between pCR patients and non-pCR patients but the SUVmax before and after nCRT and related changes had no significant difference. For the primary tumor, we observed differences in all parameters of post-nCRT PET between complete response and non-complete response patients. The MTV-T_post_ and TLG-T_post_ showed higher AUC for the prediction of pCR-T in ROC analysis compared with SUVmax-T_post_. Furthermore, the sensitivities were 100%, but the specificities were low, suggesting that these parameters were still insufficient to detect small residual tumors within primary lesions.

MRI has not been routinely used in esophageal cancer patients. However, in recent years, more studies have shown its potential in clinical staging and nCRT efficacy evaluation. MRI reached higher diagnostic accuracies than FDG-PET/CT for the detection of residual tumor in esophageal cancer patients after nCRT [25]. Adding DW-MRI to gastroscopy and endosonographic ultrasound could improve the detection of residual tumor after neoadjuvant chemoradiotherapy [26]. A systematic review implied that DW-MRI used to evaluate response to chemoradiotherapy in esophageal cancer showed variable methods and results, and a large increase in ADC after two weeks of treatment seems most predictive for a good response [27]. Previous small sample studies have verified that a treatment-induced change in ADC during the first 2–3 weeks of nCRT is most predictive for a pathological complete response to nCRT in esophageal cancer patients [12, 14, 28]. In our study, the values and increased rate of ADC of DW-MRI during nCRT were significantly different between patients with primary tumor complete response and non-complete response, the ADC values after nCRT were also different between two response groups, but the increased rate was not statistically different. The ADC values of DW-MRI during nCRT more accurately predict the pathological response than that after nCRT. Early response evaluation has a more important clinical value that could predict the treatment response early and enable individualized treatment. As the ADC value had higher specificity than PET/CT parameters,, it was supplementary to PET parameters for predicting pCR-T. The modality combining ADC_during_ and TLG-T_post_ showed the highest accuracy compared with their individual values. This result showed the great potential of multimodal imaging in identifying primary tumors response of ESCC. A recent prospective clinical study also reported similar result, but most of its population was adenocarcinoma [14].

At present, several trials have been conducted to establish a more accurate evaluation strategy for nCRT in esophageal cancer. The recently published preSANO study [19] reported that the clinical response evaluation using endoscopic ultrasonography, bite-on-bite biopsies, and fine-needle aspiration of suspicious lymph nodes was adequate for detection of locoregional residual disease, with PET-CT for detection of interval metastases. This evaluation system is now being utilized in patients with esophageal squamous cell cancer recruited from four Asian centers (pre-SINO study [29]). An additional multicenter prospective observational study developed a multimodal prediction modality, of which ctDNA and MRI were added, that accurately predicted histopathological response to nCRT (PRIDE study [30]). These studies have shown that the employment of multiple diagnostic methods may improve the accuracy of nCRT efficacy prediction.

There are some limitations of this study. First, it is a retrospective investigation at a single institution and the sample size is small. Second, although the DW-MRI examination is noninvasive and economical, the thorax remains a challenging region for MR imaging; the imaging quality should be improved and the ROIs were manually contoured in our study that might have resulted in differences in ADC measurement. The third limitation of our study is that DW-MRI and combined methods were used only for prediction of primary tumor response. One recent study [31] identified that DWI was more sensitive for assessing LN metastasis compared with PET in patients with ESCC. Since we have also found the limited value of PET/CT in predicting pN + , lymph nodes should be examined in DW-MRI in further study.

## Conclusion

The present study showed that PET/CT is useful for re-staging after nCRT and for surgical decision. Combing parameters of ^18^F-FDG PET/CT and DW-MRI had higher accuracy for predicting primary tumor response in ESCC. These findings is necessary to be validated in a prospective study with larger cohort.

## Data Availability

The data will not be shared because the ethics committee did not allow sharing of the data.

## Abbreviations

nCRT: Neoadjuvant chemoradiotherapy
EC: Esophageal cancer
ESCC: Esophageal squamous cell carcinoma
DW-MRI: Diffusion-weighted magnetic resonance imaging
^18^F-FDG-PET/CT: ^18^F-Fluorodeoxyglucose-positron emission tomography/computed tomography
SUV: Standard uptake value
MTV: Metabolic tumor volume
TLG: Total lesion glycolysis
ADC: Apparent diffusion coefficient
pCR: Pathological complete response
GTV: Gross tumor volume
CTV: Clinical target volume
PTV: Planning target volume
PD: Disease progression
UICC: Union for International Cancer Control
ROI: Region of interest
ROC: Receiver operating characteristic
